# Sudden Death Due to a Ruptured Pulmonary Trunk Aneurysm Coexisting With a Saccular Aneurysm of the Ascending Aorta in an Elderly Cigarette Smoker: A Case Report

**DOI:** 10.7759/cureus.84545

**Published:** 2025-05-21

**Authors:** Hubert Daisley, Haille D Joseph, Johann Daisley, Martina Daisley

**Affiliations:** 1 Pathology, San Fernando General Hospital, San Fernando, TTO; 2 Pathology, Scarborough General Hospital, Scarborough, TTO; 3 Anaesthesia, Scarborough General Hospital, Scarborough, TTO; 4 Emergency Medicine, Princess Alexandra Hospital, The Valley, AIA

**Keywords:** advanced age, chronic cigarette smoking, concurrent pulmonary and aortic aneurysm rupture, hemomediastinum, sudden death

## Abstract

The simultaneous occurrence of a pulmonary artery aneurysm and an aortic aneurysm is rare. Both aneurysms can have an insidious course with devastating consequences of rupture, resulting in sudden death. Elderly chronic cigarette smokers are at risk of developing both aortic and pulmonary artery aneurysms. We describe a case of a 75-year-old person whose only other comorbidity was a long history of cigarette usage, who had concurrent aortic arch aneurysm and pulmonary trunk aneurysm, with rupture of the latter causing sudden death.

## Introduction

Pulmonary artery aneurysms are uncommonly seen at autopsy [1]. The simultaneous occurrence of an ascending aortic aneurysm and a pulmonary trunk aneurysm is rare. The aetiology of pulmonary artery aneurysms is varied, with infection, trauma, and vasculitis, congenital and iatrogenic causes listed [2,3]. In this case, chronic cigarette smoking and advanced age were the only identified risk factors. We herein describe a case of an elderly person who died suddenly due to a ruptured pulmonary trunk aneurysm, with a concurrent saccular aneurysm of the ascending aorta. A review of the aetiology of pulmonary and aortic aneurysms in association with cigarette smoking is undertaken.

## Case presentation

The patient was 75 years old with no known medical conditions. The patient reportedly lived a very active life, having retired as a primary school teacher, had no admissions to hospitals for surgeries or medical conditions, and was not on any prescribed or over-the-counter medications. The patient had a history of cigarette smoking with approximately 45 pack years, until smoking ceased 20 years prior to death. The patient reportedly complained of a sore throat two days prior to death but had no other symptoms when found dead on the floor of the kitchen.

At autopsy

The body was that of a well-nourished elderly person, whose appearance was in keeping with the stated age. There were no external signs of injury. The body was anicteric, and there was mild cyanosis at the nail beds. There was cardiomegaly, with the heart weight of 580 g. There was 60 mL of hemopericardium. There was incompetence of the aortic and tricuspid valves and eccentric hypertrophy of the left ventricle with a wall thickness of 18 mm. All three coronary arteries were patent and devoid of atheroma. There were no myocardial injuries, past or present. There were no congenital defects of the heart.

**Figure 1:**
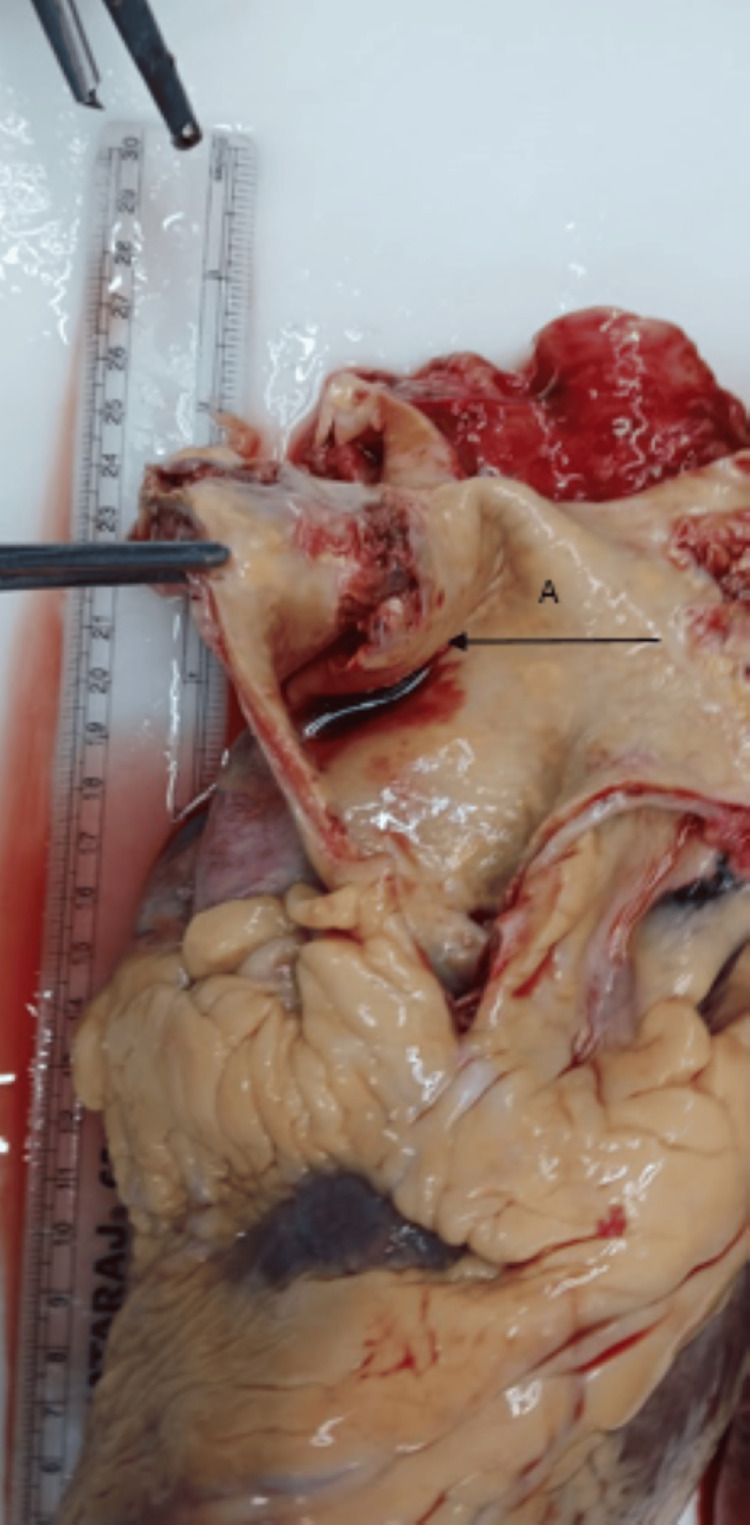
Aortic aneurysm with atheroma, focal calcification, and ulceration (arrow A)

There was a saccular aneurysm of the ascending aorta (Figure 1) beginning at its root and ascending some 8 cm with atheroma and focal severe ulcerated calcified plaques. There was no dissection of the saccular aneurysm of the aorta.

**Figure 2:**
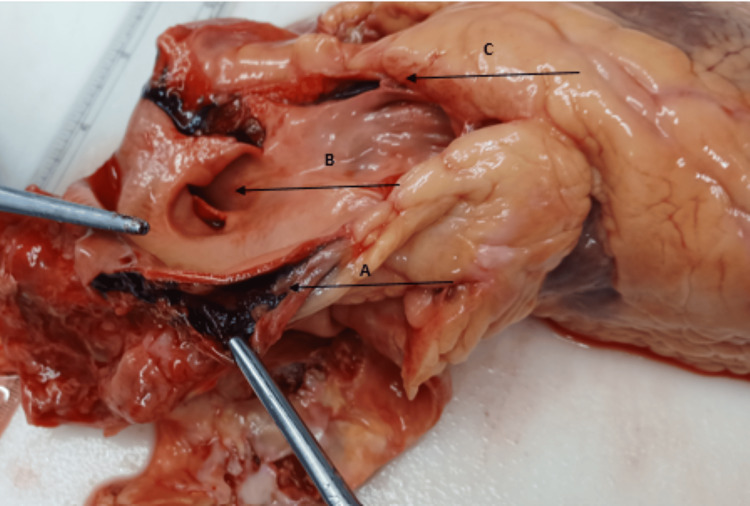
Dissection of the pulmonary trunk (A arrow), intimal tear of the pulmonary trunk (B arrow), and pulmonary valve cusp (C arrow)

There was also a saccular aneurysm of the pulmonary trunk (Figure 2) that had ruptured into the mediastinum and pericardial sac, causing haemo-mediastinum and cardiac tamponade. There was no pneumothorax or haemothorax. There was no pulmonary embolism, infarction, or consolidation. Both lungs were overweight (the left weighed 800 g and the right 840 g), oedematous, and had emphysematous changes and features of chronic obstructive airways disease. The peri-bronchial and mediastinal lymph nodes had anthracotic pigment.

The oesophagus, stomach, and the rest of the gastrointestinal system were grossly unremarkable. The internal reproductive organs were atrophic.

There was no injury to the axial or appendicular skeleton. The brain was oedematous. There were no epidural, subdural, or subarachnoid haemorrhages, nor parenchymal infarction.

Histology

**Figure 3:**
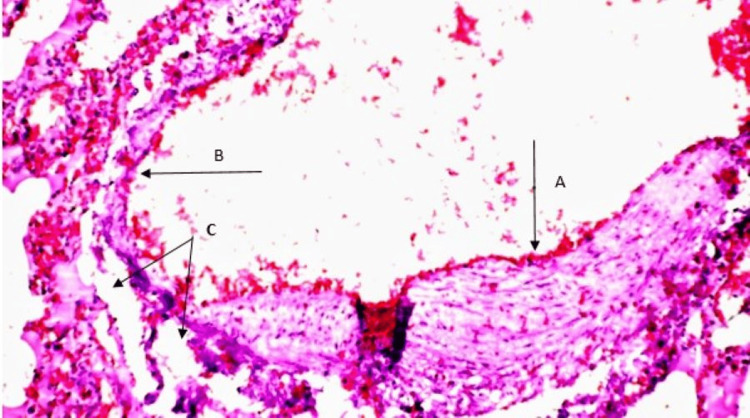
Eccentric hypertrophy of the pulmonary artery wall (A arrow), adjacent aneurysmal dilatation to paper-thinness (B arrow), and alveolar spaces (C arrow) within the lung parenchyma (H&E x200)

Lungs: There was aneurysmal dilatation of the pulmonary trunk. This dilatation continued to involve the branches of the pulmonary artery within the lung parenchyma. The walls of the pulmonary artery displayed irregular eccentric hypertrophy interrupted with aneurysmal dilatation to paper-thinness. There was pulmonary oedema and also emphysematous changes as seen in chronic obstructive pulmonary disease (Figure 3).

Heart and Aorta: There was hypertrophy of the myocardial fibres. The coronary vessels were patent and void of atheroma. There was an aneurysmal dilatation of the aorta that had atheroma and focal ulcerated calcific plaque. There was no dissection of the aorta (Figure 1).

Liver: There was chronic passive venous congestion of the liver.

## Discussion

Pulmonary artery aneurysm is an uncommon anomaly with a reported prevalence of one case being detected in 13,696 autopsies [1,2]. This prevalence report was done in the year 1947 [1], and an updated report might suggest an increasing incidence of pulmonary artery aneurysm since the advent of modern imaging techniques. The aetiology of pulmonary artery aneurysms is multifactorial [3] with infection [4], vasculitis [5], trauma [6], congenital heart disease [7], iatrogenic, and idiopathic [8,9] being the major contributing factors.

In the case in discussion, pulmonary artery hypertension from chronic obstructive airway disease (COPD), notably emphysema, caused by the individual’s long-term cigarette smoking [10,11], seems to be a contributing factor for the pulmonary artery aneurysm [11-13]. The eccentric intimal hypertrophy of the pulmonary arterial wall seen in our case is often seen in pulmonary hypertension from chronic obstructive airway disease [11,14,15].

Cigarette smoking is a major risk factor for developing saccular aneurysms of the aorta, with current smokers and former smokers having a five-fold and two-fold risk [16]. Larger aortic arch and ascending aorta diameters are seen in patients with COPD and emphysema [17], which serves as a risk factor for aortic aneurysm, especially in patients with severe lung destruction and aortic calcification [18-20].

The case in discussion is that of a former smoker, age 75 years, who had a 45-pack-year history of tobacco use, having abstained for the past 20 years prior to demise, and who was discovered to have emphysematous changes and pulmonary hypertension at autopsy. Expansion of the ascending aorta diameter from emphysema [17] due to the prolonged use of tobacco would have also contributed to the aneurysmal changes in the ascending aorta [21] seen in the case in discussion.

The simultaneous occurrence of pulmonary artery aneurysm and saccular aneurysm of the ascending aorta is extremely rare [22-25]. Our case is the fifth such case reported in the English-language literature.

Van Dinter et al reported a case of a large pulmonary artery aneurysm of the pulmonary trunk and the major branches of the pulmonary secondary to a post-dissection saccular aneurysm of the ascending aorta. Their case was that of a 70-year-old female who had several comorbidities, amongst which were smoking of 40 pack years and chronic obstructive pulmonary disease [22].

Ioakeimidis et al reported a case of a large main pulmonary artery aneurysm with dilatation of the aortic root at the level of the sinuses and proximal ascending aorta in a 77-year-old male whose history was remarkable for smoking 120 pack years [23].

Tamagno reported a case of peripheral, pulmonary artery aneurysm presenting as a solitary pulmonary nodule in a 63-year-old female who had a history of hypertension and a 40-pack-year history of cigarette smoking prior to quitting 4 years earlier [26]. Cigarette smoking is a common underlying factor in the occurrence of pulmonary and aortic aneurysms in the elderly, in our patient, and those mentioned above [22,23,26].

Our patient's comorbidities were being elderly, age 75 years, and having smoked cigarettes (45 pack years). Pulmonary hypertension and emphysema, which were discovered at autopsy, also contributed to the formation of the pulmonary artery and aortic aneurysms. Chronic cigarette smoking and COPD in the elderly seem to be the common underlying factors that predispose individuals to the occurrence of both aortic [27, 28] and pulmonary trunk aneurysms [26,29,30].

It is likely, therefore, that in the case in discussion, both pulmonary aneurysm and saccular aneurysm of the ascending aorta had their genesis in chronic cigarette smoking and in being elderly.

Chronic cigarette smoking and advanced age should be added to the aetiology of pulmonary artery aneurysm and aortic aneurysm [3].

Both pulmonary trunk and ascending aortic aneurysms are insidious and often present with devastating consequences of rupture and sudden death, as occurred in the case in discussion. Hence, it is wise to have screening chest imaging such as echocardiogram or computed tomography scan in chronic cigarette smokers, especially those in advanced ages, to detect the chronic chest diseases associated with tobacco abuse [31-34]. Early detection of these aneurysms and intervention with medical or surgical procedures may prevent catastrophic events.

## Conclusions

Concurrent aneurysms of the pulmonary trunk and ascending aorta are extremely rare and often present insidiously, particularly in elderly individuals with a history of chronic cigarette smoking. Given the catastrophic events these aneurysms can pose, early detection through routine chest imaging in high-risk patients is crucial. These pathologies should be added to the list of diseases that can occur in tobacco users. Once again, this highlights why tobacco usage should be banned.
